# The *miR-582*/*CD1B* Axis Is Involved in Regulation of Dendritic Cells and Is Associated with Clinical Outcomes in Advanced Lung Adenocarcinoma

**DOI:** 10.1155/2020/4360930

**Published:** 2020-03-17

**Authors:** Jun Guo, Hui Jin, Yanfeng Xi, Jian Guo, Yi Jin, Da Jiang

**Affiliations:** ^1^Department of Medical Oncology, Xingtai People's Hospital, Xingtai, China; ^2^Department of Oncology, Fourth Hospital of Hebei Medical University, Shijiazhuang, China; ^3^Department of Pathology, Shanxi Provincial Cancer Hospital, Taiyuan, China; ^4^Division of Thoracic Surgery, Tianjin First Central Hospital, Tianjin, China

## Abstract

The involvement of immune dysfunction in the pathogenesis of lung cancer has been extensively studied. However, the potential molecular mechanisms through which the tumor immune response affects drug resistance are still unclear. Accordingly, in this study, we evaluated deviations in the immune cell landscape among patients with different stages of lung adenocarcinoma to identify key microRNAs and their targets associated with patient outcomes. CIBERSORT was used for estimating the proportions of immune cells in various lung tissues. Significantly different adaptive and innate immune cell types, including memory B cells, CD8+ T cells, resting dendritic cells, and resting mast cells, were selected. Comparative studies and survival analyses were carried out. We found that potential genes and microRNAs involved in immune responses were associated with patient outcomes. Specifically, *miR-582/CD1B*, which are involved in resting and activated dendritic cells, may be potential novel biomarkers for immunotherapy. An independent dataset of miRNA microarray profiles was used to validate the expression of mature *miR-582-5p* in patients with advanced lung adenocarcinoma. Alternative treatments, including immunotherapies and chemotherapy, are urgently needed to improve outcomes in patients with lung cancer. Thus, our findings could provide insights into the selection of novel microRNAs targeting immune genes and could improve the efficacy of immunotherapy by disrupting tumor function and promoting immune infiltration in patients with advanced lung adenocarcinoma.

## 1. Introduction

Lung adenocarcinoma (LADC) is a major cause of cancer-related death worldwide, accounting for approximately 40% of non-small-cell lung cancers (NSCLCs) [1, 2]. In early stages, patients with nonmetastatic lung cancer typically undergo surgical resection. However, patients with metastatic or advanced stage disease are treated with chemotherapy alone or in combination with radiation [3]. Although many innovative therapies, including immunotherapies and molecular targeted therapies, have been developed, the survival rate of patients with LADC is still low because of histological subtype tumor heterogeneity, poor understanding of disease pathogenesis, and drug resistance. Therefore, additional molecular characterization of the LADC landscape could help researchers and clinicians to identify novel biomarkers or molecular targets, design novel therapeutic strategies, and improve patient outcomes [4].

In the past decade, the import roles of the tumor microenvironment (TME) in the initiation and progression of primary and secondary lung carcinoma have been uncovered, and the TME has been recognized as a target-rich environment for novel anticancer agents [5–7]. Several approved drugs targeting different biomarkers in the TME have been used in the clinical setting; these include immune checkpoint inhibitors and vascular endothelial growth factor inhibitors [8]. Newman et al. developed the *in silico* tool CIBERSORT, which can be used to quantify 22 immune cell types using 547 gene expression profiles from various tissues [9]. This approach is easier and more convenient than traditional approaches for identification of immune cell-based prognostic and therapeutic markers after stratification into molecular subtypes. Previous studies have also evaluated the roles of innate and adaptive immune dysfunction in the lung TME, which could promote or suppress tumor activities and affect clinical outcomes [10–12].

During the adaptive immune response, various subtypes of T cells, particularly CD4+ and CD8+ T cells, infiltrate tumors and mediate responses to immune checkpoint inhibition. Markowitz et al. reported that the depletion of CD4+ and/or CD8+ T cells combined with an anti-programmed cell death 1 (PD1) antibody reduces the therapeutic efficacy of the PD1 blockade in a KRAS-driven mouse model of NSCLC [13]. Tumor infiltration of B cells also plays key roles in the TME. Germain et al. showed that B cells and CD4+ T cells reside in tertiary lymphoid structures and are associated with an improved prognosis in patients with NSCLC [14].

During the innate immune response, dysfunction of dendritic cells (DCs), neutrophils, and natural killer (NK) cells has also been reported in studies of lung cancer. DCs fail to stimulate T cells because of upregulation of the coinhibitory molecule CD276 in patients with lung cancer [15]. Moreover, transforming growth factor-*β* (TGF*β*), which induces the differentiation of CD4+ T cells to suppress T cell proliferation, can be produced by DCs [16]. The function of neutrophils is also complex; these cells can not only promote carcinogenesis through angiogenesis and metastasis but also limit the growth of tumors through production of antitumor and cytotoxic mediators [17, 18]. In the lung TME, TGF*β* regulates NK cell responses by mediating the polarization of NK cells towards a proangiogenic phenotype [19]. Taken together, these studies suggest that detecting dysfunction of innate and adaptive immunity in the occurrence and development of lung cancer is necessary for fully elucidating the potential molecular mechanisms.

MicroRNAs (miRNAs) are small noncoding RNAs of approximately 20–24 nucleotides. These molecules have recently been shown to modulate gene expression via posttranscriptional regulation of mRNA and are important biomarkers of tumor suppressors, oncogenes, diagnosis, and prognosis. miRNAs affect immune escape, leading to the generation of a TME favoring tumor growth and progression [20]. Furthermore, miRNAs have also been shown to affect the regulation of immune checkpoints, including PD1 and PD1 ligand [21–23]. However, the mechanisms through which miRNAs regulate immune responses are still unclear.

Accordingly, in this study, we used CIBERSORT to estimate the proportions of different immune cells in LADC samples with different TNM stages and then examined the roles of miRNAs and their targets in determining survival and patient outcomes in patients with LADC. Our findings provided insights into the applications of immunotherapies in patients with LADC.

## 2. Materials and Methods

### 2.1. Datasets and Preprocessing

First, gene expression profiles and miRNA expression profiles from 495 LADC samples were downloaded from the UCSC Xena platform [24]. The proportions of different immune cells among all samples were estimated based on LM22 signature files using CIBERSORT [9], an analytical tool that can accurately quantify the relative levels of distinct immune cell types within a complex gene expression mixture. To characterize and quantify each immune cell subtype, CIBERSORT used gene expression signatures consisting of 547 genes (LM22 files). From the results of CIBERSORT, 318 samples that met the requirements of CIBERSORT *p* value less than 0.05 were selected. Among these samples, 315 samples (177, 77, 48, and 13 samples with TNM stages 1–4, respectively) were selected for further analysis of clinical outcomes (i.e., overall survival (OS)) and had paired miRNA/RNA sequencing data. All samples were divided into two groups according to TNM stage, i.e., early stage (TNM stages 1 and 2) and advanced stage (TNM stages 3 and 4). An independent miRNA profile microarray (GSE48414) of LADC with different stages and an independent gene profile microarray (GSE31210) of LADC with OS were used for validation [25, 26].

### 2.2. Analysis of Differentially Expressed Genes (DEGs) and miRNAs

DEGs and differentially expressed miRNAs were identified between different lung tissues with the threshold of absolute fold change greater than 1.5 and adjusted *p* value less than 0.05 using R package “limma.”.

### 2.3. Identification of miRNA/Target Gene Pairs

Candidate pairs of miRNA/target gene were predicted using the prediction algorithm MirTarget [27]. MirTarget predicted targets with scores between 50 and 100; higher scores indicated higher confidence of prediction. A predicted target with a prediction score of greater than 80 was considered most likely to be real; accordingly, we set this as our cutoff for selection. Stem-loop miRNAs were identified in The Cancer Genome Atlas (TCGA), and mature miRNAs were identified in MirTarget; the miRNA identification information was downloaded from miRBase (V22.1), and stem-loop miRNAs were mapped into mature miRNAs [28]. The regulation of miRNAs and target genes was visualized by Cytoscape (Version 3.7.1) [29]. The regulation of *miR-582* and *CD1B* was predicted by TargetScanHuman (Release 7.2) [30].

### 2.4. Survival Analysis

COX Hazards (COXPH) regression was carried out to assess whether proportions of immune cells from CIBERSORT and related gene expression levels were associated with patient outcomes. Proportions of immune cells from CIBERSORT and expression values of genes consistently identified were grouped into high and low categories based on median values. Kaplan-Meier survival analysis and log-rank tests were used. All statistics were calculated using R language (Version 3.5.2).

## 3. Results

### 3.1. Data Processing

**Figure 1:**
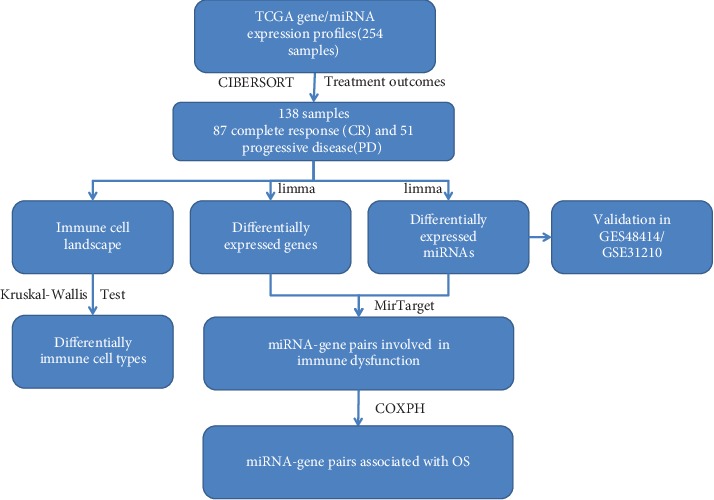
Workflow of integrative analysis of the regulation of miRNA-target in LADC.

Gene expression profiles from 495 LADC samples were downloaded from the UCSC Xena platform [24]. The proportions of different immune cells for all samples were then estimated based on LM22 signature files using CIBERSORT [9]. From the results of CIBERSORT, 318 samples were selected, among which 315 were used for further analysis of clinical outcomes (OS) and had paired microRNA/RNA sequencing data (details are given in Supplementary Materials [Supplementary-material supplementary-material-1]). All samples were then divided into early and advanced stage LADC. The workflow is shown in Figure 1.

### 3.2. Immunity Cell Comparison

**Figure 2:**
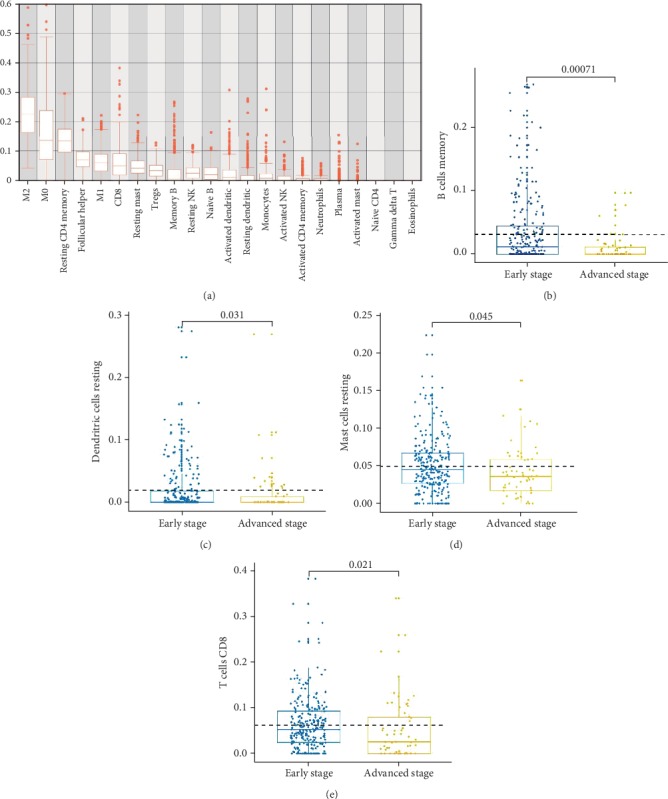
Fractions of immune cells in various LADC samples. Fractions of 22 immune cells in all LADC samples calculated by CIBERSORT are shown in (a). CIBERSORT immune cell fractions were determined for each sample; each dot represents one sample. Mean values and standard deviations for each cell subset, including B memory cells (b), resting dendritic cells (c), resting mast cells (d), and CD8+ T cells (e), were calculated for each sample group and compared using Kruskal-Wallis tests.

CIBERSORT was used to estimate the fractions of different immune cells during data preprocessing (Supplementary Materials [Supplementary-material supplementary-material-1]). As shown in Figure 2(a), M2 macrophages, M0 macrophages, CD4+ resting memory T cells, T follicular helper cells, and M1 macrophages were the five most common immune cell fractions in LADC, and the sum of their mean proportions was 67.16% for all clinical subgroups. The mean and standard deviation values of cell fractions in all 22 cell types are described in Table 1. Comparative studies were carried out to reveal differences between different stages. Four immune cell types, including memory B cells (*p* = 7.09*E* − 4), CD8+ T cells (*p* = 2.15*E* − 2), resting DCs (*p* = 3.15*E* − 2), and resting mast cells (*p* = 4.51*E* − 2), were altered significantly between patients with early stage and advanced stage LADC (Figures 2(b)–2(e)). Interestingly, the fractions of all four of these cell types were lower in patients with advanced stage disease than in patients with early stage disease. Differences in cell fractions between the four stages are shown in Supplementary [Supplementary-material supplementary-material-1]. These results provided evidence of the dysfunctional immune response in advanced LADC.

### 3.3. miRNA/Target Gene Pairs Involved in the Immune Response

In order to determine the molecular mechanisms of different fractions of immune cells and identify candidate genes, miRNA/target gene pairs were predicted. First, stem-loop miRNAs expressed (RSEM > 0) in at least half of LADC samples were selected. Second, the selected miRNAs were transformed into mature miRNAs by miRBase [28]. Finally, the regulation of 547 immune genes contained in CIBERSORT by mature miRNAs was predicted by MirTarget [27]. In total, 3935 pairs of miRNAs/target genes, including 554 stem-loop miRNAs (802 mature miRNAs) and 413 genes, were selected.

**Figure 3:**
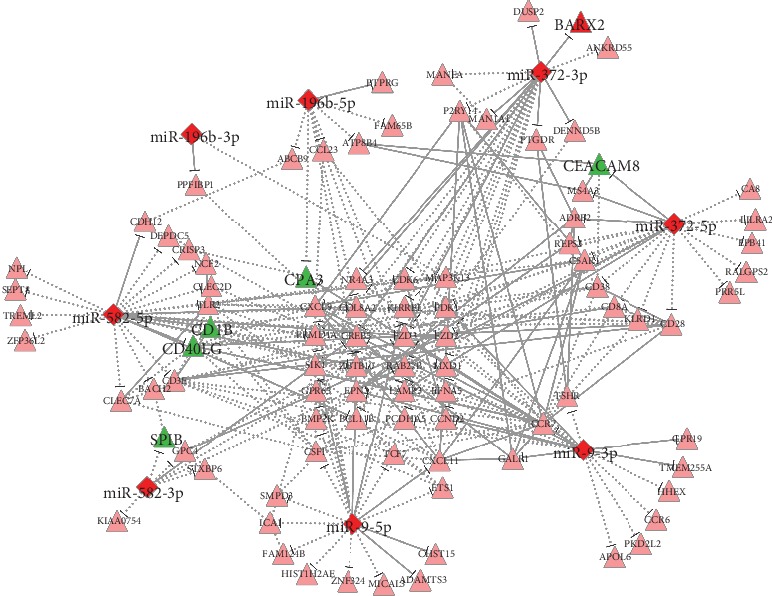
miRNA and miRNA/target gene interaction networks. Diamonds represent miRNAs, and triangles represent immune genes targeted by miRNAs. Red indicates upregulated, and green indicates downregulated. The regulatory relationships between miRNA and genes were from MirTarget, and the interactions between genes were from String database. The solid line indicates that the relationship had high confidence (miRNA/gene prediction score > 80 or PPI combined score > 0.7), and the dotted line indicates that the relationship had low confidence.

DEGs and differentially expressed miRNAs were then identified. Among 547 immune genes, 40 genes were downregulated, and three genes were upregulated. Most of the DEGs were involved in memory B cells and CD8+ T cells, and these results were consistent with differences in cell fractions estimated by CIBERSORT. Moreover, six miRNAs (*miR-582*, *miR-372*, *miR-196b*, *miR-9-1*, *miR-9-2*, and *miR-9-3*) were upregulated, and no miRNAs were downregulated. The String database was used to find interactions between DEGs (combined score > 0.7), and an miRNA/gene interaction network was then constructed (Figure 3 and Supplementary Materials [Supplementary-material supplementary-material-1]).

### 3.4. Influence of mRNA and miRNA Expressions on OS

**Figure 4:**
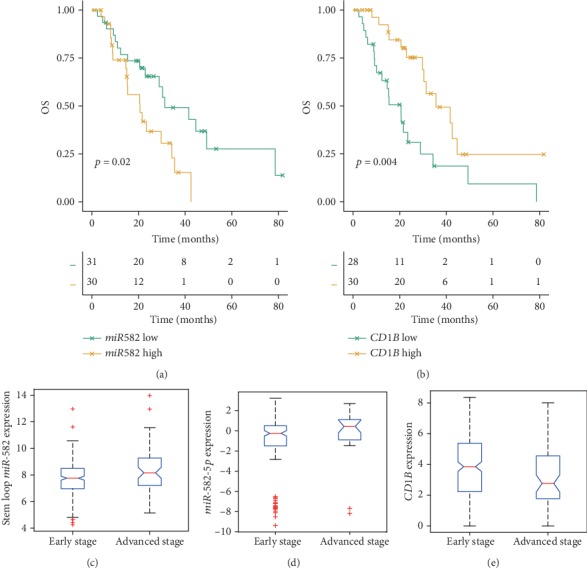
Association of *miR-582/CD1B* with OS. Patients were stratified into high and low categories based on the median expression level of *miR-582* (or *CD1B*) for Kaplan-Meier survival analysis by OS, and the results are shown (a, b). The different expression levels of *miR-582* and *CD1B* are shown (c, e). The expression levels of mature *miR-582-5p* in LADC samples from various stages were determined using the independent miRNA profile microarray GSE48414 (d).

As previously described, we observed significant differences in immune cell compositions between early and advanced stage LADC. Immune cell migration and/or retention in tumors can affect OS and/or recurrence-free survival [13]. Therefore, we hypothesized that genes and miRNAs involved in these immune cells could be significantly associated with OS. COXPH analysis identified 35 DEGs and 1 differentially expressed miRNA significantly associated with OS (Table 2). The upregulated miRNA *miR-582* was significantly associated with OS (*p* = 1.34*E* − 4, hazard ratio (HR), 95% confidence interval (CI): 1.5 (1.22–1.85)) in advanced stage disease. Kaplan-Meier analyses and log-rank tests suggested that *miR-582* was significantly negatively associated with OS (Figure 4(a)). Moreover, *miR-582* regulated the DEG *CD1B*, which was identified as a biomarker of resting and activated DCs. *CD1B* has been shown to be related to the major histocompatibility complex proteins and mediates the presentation of primarily lipid and glycolipid antigens of self or microbial origin to T cells. COXPH analysis showed that *CD1B* was significantly associated with OS in early stage disease (*p* = 2.39*E* − 2, HR (95% CI): 0.868 (0.767–0.982)), advanced stage disease (*p* = 4.25*E* − 2, HR (95% CI): 0.828 (0.69–0.995)), and all samples (*p* = 3.22*E* − 4, HR (95% CI): 0.831 (0.751–0.92)). Significant *CD1B* results of Kaplan-Meier analyses and log-rank test are shown in Figure 4(b). The expression levels of *miR-582* and *CD1B* are shown in Figures 4(c) and 4(e). *CD1B* was downregulated, whereas *miR-582* was upregulated in LADC samples from patients with advanced stage disease.

**Figure 5:**
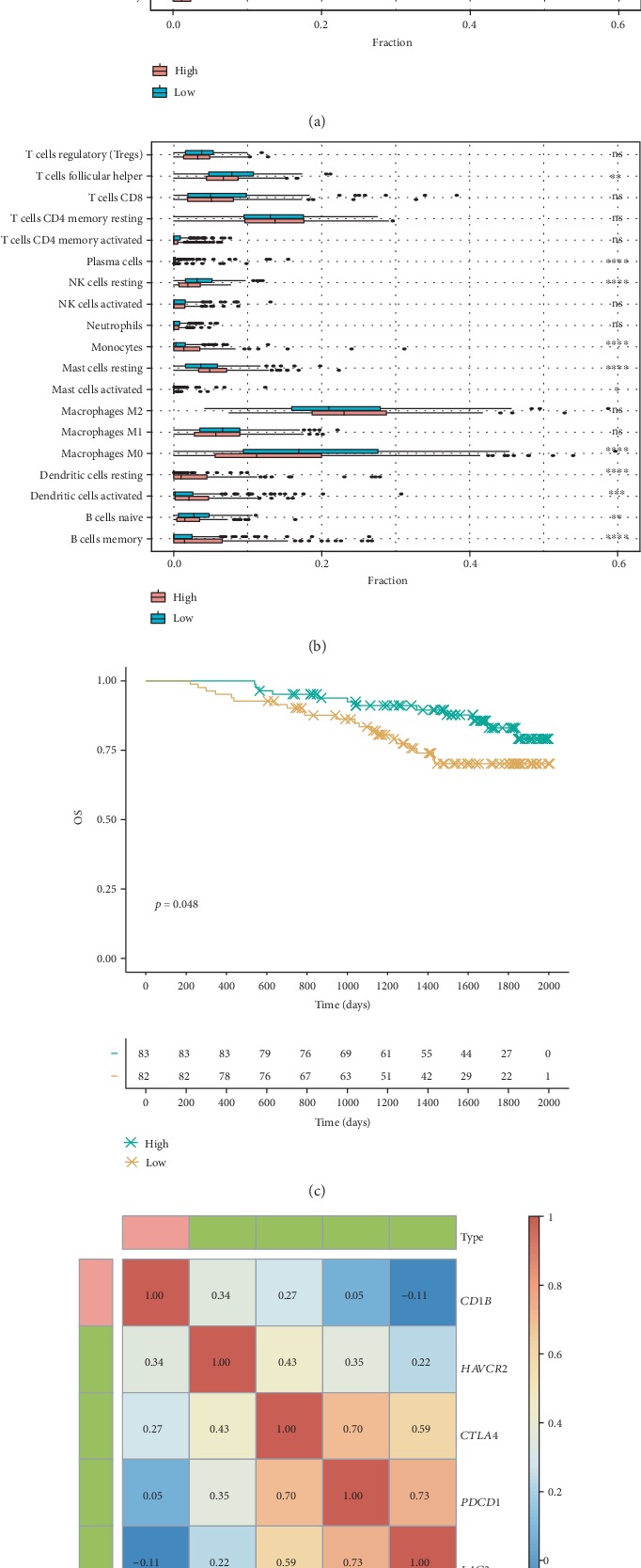
Relationships between *miR-582* and *CD1B* expression and immune cell types. Patients were stratified according to *miR-582* and *CD1B* expression. The different fractions of immune cell subtypes were then compared using Kruskal-Wallis tests (a, b). Patients were stratified into high and low categories based on the median expression level of *CD1B* for Kaplan-Meier survival analysis by OS, and the results were determined based on the independent gene profile microarray GSE31210 (c). Pearson correlation coefficients between *CD1B* and various checkpoint molecules (*PDCD1*, *CTLA4*, *LAG3*, and *HAVCR2*) were calculated (d). The regulation of *miR-583* and *CD1B* was predicted by TargetScanHuman (e).

Furthermore, other miRNA/target predication methods and independent miRNA/gene expression profiles were used to validate our results. First, the regulation of *miR-582* and *CD1B* was also validated (Figure 5(e)) using TargetScanHuman [30]. Second, to investigate the expression levels of mature *miR-582-5p* produced by stem-loop *miR-582*, an independent miRNA profile microarray (GSE48414) was used (Figure 4(d)). The results showed that *miR-582-5p* was upregulated (fold change = 2.06) in advanced LADC. Moreover, an independent gene profile microarray (GSE31210) of LADC showed that *CD1B* was significantly associated with OS (*p* = 0.048; Figure 5(c)). Taken together, these results suggested that *miR-582/CD1B* may play key roles in the dysfunction of DCs and could be associated with clinical outcomes in advanced LADC.

## 4. Discussion

The efficacy of treatments for lung cancer is limited by a lack of early detection methods and the acquisition of drug resistance. Tumor cells can distort host immune checkpoints in various ways to escape immune responses and promote the development and progression of lung cancer [31]. Currently, researchers are focusing on checkpoints for limiting the activity of T cells by cytotoxic T lymphocyte antigen- (CTLA-) 4 in early stage disease and PD1 (and/or PD-L1) in advanced stage disease [32]. The use of monoclonal antibodies and other blockers to block these immune checkpoints has been reported to exert beneficial effects in patients with NSCLC in clinical trials [33]. Thus, the identification of novel and effective immunotherapeutic targets is urgently required.

miRNAs play key roles as potential biomarkers for resistance or sensitivity to chemotherapeutic drugs in lung cancer. For example, *miR-200* has been found to be associated with high PD-L1 expression, which is involved in intratumoral immunosuppression by targeting ZEB [22]. Additionally, Boldrini et al. reported that *miR-33a-5p* is highly expressed in LADC; negatively related to PD1, PD-L1, and CTLA-4 expression; and positively associated with an improved prognosis [23]. Furthermore, the *miR-197*/CDC28 protein kinase regulatory subunit 1B/signal transducer and activator of transcription 3 regulatory network mediates PD-L1 expression and is associated with outcomes in patients with NSCLC [21]. Thus, the discovery of novel miRNAs and their targeted immune genes (or immune checkpoints) may facilitate the development of novel beneficial therapeutic modalities for patients with lung cancer, particularly for those with drug-resistant phenotypes.

In this study, CIBERSORT was applied to assess differential immune cell fractions between early and advanced stage LADC. Notably, memory B cells, CD8+ T cells, resting DCs, and resting mast cells were significantly reduced in patients with advanced stage cancer. There were 43 DEGs and six miRNAs that were identified. Most of the DEGs were downregulated and were involved in memory B cells and CD8+ T cells, and all of the miRNAs were upregulated. COXPH analysis suggested that the fraction of resting DCs was significantly related with OS. Furthermore, *miR-582* was significantly associated with OS by targeting *CD1B*. Recently, a phase I trial of patients with advanced NSCLC suggested that intratumoral vaccination with autologous DCs could increase infiltration of CD8+ T cells into tumors and increase the expression levels of PD-L1 [34].

In our study, we stratified patients according to *miR-582* and *CD1B* expression and showed that patients with higher *CD1B* expression showed significantly increased fractions of resting mast cells, monocytes, memory B cells, and activated and resting DCs but significantly decreased fractions of follicular helper T cells, resting NK cells, M0 macrophages, and naïve B cells (Figures 5(a) and 5(b)). These results suggested that *CD1B* may be involved in the biological processes of the immune system. Additionally, we identified several checkpoint-related molecules, including *PDCD1*, *CTLA4*, *LAG3*, and *HAVCR2*, and showed that *CD1B* was significantly positively correlated with *HAVCR2* and *CTLA4* (Figure 5(d)).

The use of miRNAs as therapeutic agents is still being investigated. The expression levels of oncogenic (or tumor-suppressive) miRNAs can be changed using small interfering RNA, miRNA mimics, and small molecule inhibitors of miRNAs. A recent phase I clinical trial showed that *miR-34a*-loaded liposomes (MRX34) significantly decreases the expression of PD-L1 [35]. In addition, a combination of MRX34 and radiotherapy can enhance the CD8+ cell count and reduce tumor infiltration by macrophages and regulatory T cells [35]. Thus, the use of miRNAs as therapeutic agents may become realistic in the future. Importantly, in this study, we identified six upregulated miRNAs (*miR-582*, *miR-372*, *miR-196b*, *miR-9-1*, *miR-9-2*, and *miR-9-3*) in the comparison of early and advanced stage LADC. Among these miRNAs, *miR-582* has been reported to promote tumorigenesis by targeting phosphatase and tensin homolog in colorectal cancer [36]. Moreover, *miR-196b* promotes cell migration and invasion by targeting *FOXP2* in hepatocellular carcinoma and regulates self-renewal, differentiation, and transformation by targeting *HOXC8* in breast cancer stem cells [37, 38]. *miR-9* has an inhibitory role in papillary thyroid cancer by targeting *BRAF* and reduces metastatic behavior in triple-negative breast cancer by targeting *NOTCH1* [39, 40]. However, the roles of these miRNAs in lung cancer and their potential therapeutic applications have not been evaluated.

The goal of this study was to identify variant immune cell fractions in different stages of LADC and elucidate key miRNAs and their targeting immune genes. However, there were some limitations to this study. First, the patient sample size, particularly for the group of advanced LADC samples, was not large. Second, TCGA datasets only contained tumor samples. Thus, analysis of paired adjacent tissues is required in order to fully understand changes in the expression levels of miRNAs and immune genes occurring in healthy tissues versus advanced LADC. Third, additional approaches and immune-related genes could be used for estimating the fractions and activation of various immune cells. Further studies should also evaluate whether the cutoff values set in this study could have an effect on prediction of the regulation of miRNAs and targets. What is more, laboratory studies should be performed to confirm these results. Despite these limitations, our findings provided important insights into the roles of miRNAs in the development, progression, and treatment of LADC.

## Figures and Tables

**Table 1:** The cell fractions in different lung cancer samples calculated by CIBERSORT.

Cell type	Wilcoxon *p* value	Mean (early)	Mean (advanced)	Std (early)	Std (advanced)
B cells naive	1.10*E* − 01	2.66	3.16	2.78	2.87
B cells memory	7.09*E* − 04	3.52	1.17	5.51	2.30
Plasma cells	9.32*E* − 01	0.51	0.24	1.91	0.68
T cells CD8	2.15*E* − 02	6.43	5.33	5.82	6.80
T cells CD4 memory resting	2.01*E* − 01	13.71	12.64	5.89	6.14
T cells CD4 memory activated	5.80*E* − 01	0.70	0.90	1.37	1.65
T cells follicular helper	9.49*E* − 01	7.33	7.46	3.83	4.33
T cells regulatory (Tregs)	4.18*E* − 01	3.58	3.34	2.52	2.56
NK cells resting	3.55*E* − 01	2.83	3.16	2.40	2.49
NK cells activated	5.88*E* − 01	1.06	0.87	1.78	1.40
Monocytes	3.51*E* − 01	1.88	1.54	3.24	2.45
Macrophages M0	1.03*E* − 01	16.04	18.73	12.28	12.79
Macrophages M1	7.35*E* − 01	6.58	6.75	4.36	5.30
Macrophages M2	2.13*E* − 01	22.81	24.47	8.59	10.25
Dendritic cells resting	3.14*E* − 02	2.00	1.47	4.11	4.04
Dendritic cells activated	5.28*E* − 02	2.50	3.66	3.89	4.51
Mast cells resting	4.51*E* − 02	5.11	4.18	3.72	3.44
Mast cells activated	7.23*E* − 01	0.18	0.18	1.00	0.86
Neutrophils	3.63*E* − 01	0.50	0.66	0.93	1.01

**Table 2:** Differentially expressed genes and miRNA associated with OS.

Symbol	FC	HR (95% CI)	COX_P
ACHE	-0.69	0.866 (0.779-0.963)	7.79*E* − 03
AMPD1	-0.64	0.888 (0.799-0.987)	2.77*E* − 02
BARX2	0.65	1.12 (1.02-1.24)	2.21*E* − 02
BLK	-0.8	0.874 (0.794-0.963)	6.38*E* − 03
CCL17	-0.67	0.893 (0.816-0.978)	1.40*E* − 02
CD19	-0.85	0.879 (0.802-0.962)	5.17*E* − 03
CD1B	-0.74	0.831 (0.751-0.92)	3.22*E* − 04
CD1E	-0.68	0.85 (0.775-0.933)	5.30*E* − 04
CD22	-0.7	0.843 (0.746-0.952)	5.89*E* − 03
CD40LG	-0.63	0.795 (0.691-0.914)	1.26*E* − 03
CD79A	-0.73	0.872 (0.781-0.974)	1.48*E* − 02
CD79B	-0.6	0.814 (0.709-0.934)	3.38*E* − 03
CEACAM8	-0.9	0.803 (0.695-0.927)	2.35*E* − 03
CPA3	-0.62	0.879 (0.797-0.968)	8.81*E* − 03
CR2	-0.99	0.889 (0.825-0.958)	1.94*E* − 03
CTSG	-0.87	0.86 (0.782-0.946)	1.90*E* − 03
CXCR5	-0.69	0.863 (0.765-0.974)	1.68*E* − 02
FCER2	-0.79	0.878 (0.797-0.967)	8.44*E* − 03
FCRL2	-0.74	0.86 (0.771-0.959)	6.65*E* − 03
GRAP2	-0.59	0.798 (0.679-0.937)	5.85*E* − 03
HDC	-0.63	0.863 (0.767-0.971)	1.39*E* − 02
HLA-DOB	-0.64	0.774 (0.664-0.902)	1.04*E* − 03
HPGDS	-0.68	0.823 (0.74-0.915)	3.22*E* − 04
IL5RA	-0.62	0.878 (0.788-0.979)	1.88*E* − 02
LY9	-0.61	0.767 (0.656-0.897)	8.93*E* − 04
MS4A1	-0.99	0.879 (0.811-0.953)	1.76*E* − 03
MS4A2	-0.65	0.851 (0.763-0.949)	3.52*E* − 03
NCR3	-0.66	0.819 (0.713-0.94)	4.60*E* − 03
SKA1	0.59	1.27 (1.1-1.47)	1.53*E* − 03
SPIB	-0.84	0.858 (0.768-0.958)	6.52*E* − 03
STAP1	-0.84	0.762 (0.67-0.866)	2.99*E* − 05
TNFRSF13B	-0.87	0.861 (0.775-0.957)	5.52*E* − 03
TRAT1	-0.62	0.875 (0.767-0.999)	4.87*E* − 02
VPREB3	-0.67	0.861 (0.769-0.963)	8.70*E* − 03
ZAP70	-0.62	0.86 (0.753-0.982)	2.59*E* − 02
hsa-mir-582	0.62	1.22 (1.05-1.4)	8.09*E* − 03

## Data Availability

The gene expression profiles, miRNA expression profiles, phenotypes and survival data of the 495 TCGA LADC samples used to support the findings of this study have been deposited in the UCSC Xena platform (https://xenabrowser.net/datapages/) and named “GDC TCGA Lung Adenocarcinoma (LUAD)”. The independent miRNA profile GSE48414 and the independent gene profile GSE31210 were obtained from the gene expression omnibus (https://www.ncbi.nlm.nih.gov/geo/).

## Supplementary Materials

Supplementary Materials Supplementary Materials 1: the clinical information of 315 samples. Supplementary Materials 2: the fractions of different immune cells of 315 samples. Supplementary Materials 3: the details of miRNA/gene interaction network. Supplementary Figure S1: differences in cell fractions between the four stages.
