# Supplementary material for: The miR-582/CD1B Axis Is Involved in Regulation of Dendritic Cells and Is Associated with Clinical Outcomes in Advanced Lung Adenocarcinoma
Source: Biomed Res Int. 2020 Mar 17;2020:4360930. doi: 10.1155/2020/4360930 (PMC7103041; doi:10.1155/2020/4360930)
Supplement: Supplementary Materials — 1: the clinical information of 315 samples. Supplementary Materials 2: the fractions of different immune cells of 315 samples. Supplementary Materials 3: the details of miRNA/gene interaction network. Supplementary Figure S1: differences in cell fractions between the four stages. [file 4360930.f1.zip › Supplementary meterials3.docx]

| Node1 | Node2 | Score | Type | Node1 | Node2 | Score | Type |
| --- | --- | --- | --- | --- | --- | --- | --- |
| CDK6 | CCND2 | HC^1^ | PP^2^ | miR582-5p | CDH12 | HC | M2G^4^ |
| CXCL9 | CXCL11 | HC | PP | miR582-5p | CD40LG | HC | M2G |
| CD3E | CD28 | HC | PP | miR582-5p | BCL11B | HC | M2G |
| CXCL9 | CCR7 | HC | PP | miR582-3p | CREB5 | HC | M2G |
| CD8A | CD3E | HC | PP | miR9-3p | GALR1 | HC | M2G |
| CXCL11 | CCR7 | HC | PP | miR372-5p | LAMP3 | HC | M2G |
| CD8A | CD28 | HC | PP | miR582-5p | CD1B | HC | M2G |
| CLEC7A | TLR2 | HC | PP | miR372-5p | CEACAM8 | HC | M2G |
| CD1B | CD3E | HC | PP | miR372-5p | ADRB2 | HC | M2G |
| TSHR | ADRB2 | HC | PP | miR372-3p | PTGDR | HC | M2G |
| MS4A3 | CEACAM8 | HC | PP | miR372-3p | NR4A3 | HC | M2G |
| ADRB2 | PTGDR | HC | PP | miR372-3p | MAN1A1 | HC | M2G |
| CXCL9 | C5AR1 | HC | PP | miR372-3p | FZD3 | HC | M2G |
| C5AR1 | CCR7 | HC | PP | miR372-3p | FRMD4A | HC | M2G |
| C5AR1 | CXCL11 | HC | PP | miR372-3p | DUSP2 | HC | M2G |
| CXCL9 | P2RY14 | HC | PP | miR372-3p | DENND5B | HC | M2G |
| FZD3 | FZD2 | HC | PP | miR372-3p | CREB5 | HC | M2G |
| ATP8B4 | MS4A3 | HC | PP | miR372-3p | BARX2 | HC | M2G |
| P2RY14 | CCR7 | HC | PP | miR196b-5p | PTPRG | HC | M2G |
| C5AR1 | GALR1 | HC | PP | miR196b-3p | PPFIBP1 | HC | M2G |
| P2RY14 | CXCL11 | HC | PP | miR372-5p | MS4A3 | HC | M2G |
| C5AR1 | P2RY14 | HC | PP | miR9-5p | CHST15 | HC | M2G |
| TSHR | PTGDR | HC | PP | miR9-5p | KIRREL | HC | M2G |
| P2RY14 | GALR1 | HC | PP | miR9-3p | TMEM255A | HC | M2G |
| GALR1 | CCR7 | HC | PP | miR9-3p | PDK1 | HC | M2G |
| REPS2 | EPN2 | HC | PP | miR9-5p | SIK1 | HC | M2G |
| CXCL11 | GALR1 | HC | PP | miR9-5p | CREB5 | HC | M2G |
| REPS2 | ADRB2 | HC | PP | miR9-3p | GPR19 | HC | M2G |
| CXCL9 | GALR1 | HC | PP | miR9-5p | CXCL11 | HC | M2G |
| EPN2 | ADRB2 | HC | PP | miR9-5p | ADAMTS3 | HC | M2G |
| ATP8B4 | CEACAM8 | HC | PP | miR372-3p | P2RY14 | LC | M2G |
| CD40LG | CD28 | HC | PP | miR9-5p | FRMD4A | LC | M2G |
| CD28 | CCR7 | HC | PP | miR372-3p | MXD1 | LC | M2G |
| GPC4 | FZD2 | HC | PP | miR372-3p | SIK1 | LC | M2G |
| NCF2 | TLR2 | HC | PP | miR372-3p | PCDHA5 | LC | M2G |
| CD28 | CD38 | HC | PP | miR9-5p | FAM124B | LC | M2G |
| CD40LG | CCR7 | HC | PP | miR372-3p | RAB27B | LC | M2G |
| CD3E | CCR7 | LC^3^ | PP | miR372-3p | MANEA | LC | M2G |
| CCR7 | CD38 | LC | PP | miR372-5p | BMP2K | LC | M2G |
| CD1B | LAMP3 | LC | PP | miR372-3p | GPR65 | LC | M2G |
| FZD3 | GPC4 | LC | PP | miR372-5p | C5AR1 | LC | M2G |
| CD40LG | CD38 | LC | PP | miR372-5p | CA8 | LC | M2G |
| GPR29 | CD28 | LC | PP | miR372-5p | CD28 | LC | M2G |
| CD40LG | TLR2 | LC | PP | miR372-5p | CD38 | LC | M2G |
| BCL11B | TCF7 | LC | PP | miR372-5p | CD8A | LC | M2G |
| FAM124B | ICA1 | LC | PP | miR9-5p | CXCL9 | LC | M2G |
| CXCL9 | CSF1 | LC | PP | miR372-5p | CXCL9 | LC | M2G |
| CXCL9 | GPR29 | LC | PP | miR372-5p | EPB41 | LC | M2G |
| C5AR1 | TLR2 | LC | PP | miR9-5p | ETS1 | LC | M2G |
| CXCL9 | TLR2 | LC | PP | miR372-3p | BCL11B | LC | M2G |
| CD1B | CCR7 | LC | PP | miR9-5p | TCF7 | LC | M2G |
| TLR2 | CCR7 | LC | PP | miR196b-5p | ABCB9 | LC | M2G |
| CSF1 | TLR2 | LC | PP | miR196b-5p | ATP8B4 | LC | M2G |
| CCL23 | CXCL9 | LC | PP | miR196b-5p | CCL23 | LC | M2G |
| LAMP3 | CCR7 | LC | PP | miR196b-5p | COL8A2 | LC | M2G |
| C5AR1 | CLEC7A | LC | PP | miR196b-5p | CPA3 | LC | M2G |
| CD28 | TLR2 | LC | PP | miR196b-5p | FAM65B | LC | M2G |
| CD1B | CD38 | LC | PP | miR196b-5p | FZD3 | LC | M2G |
| GPR29 | CXCL11 | LC | PP | miR9-5p | SMPD3 | LC | M2G |
| GPR29 | CD38 | LC | PP | miR372-3p | LAMP3 | LC | M2G |
| CCL23 | CXCL11 | LC | PP | miR372-3p | ANKRD55 | LC | M2G |
| CD3E | CD38 | LC | PP | miR9-5p | GPR65 | LC | M2G |
| KLRD1 | CD28 | LC | PP | miR372-3p | CCND2 | LC | M2G |
| CD40LG | CD1B | LC | PP | miR372-3p | CDK6 | LC | M2G |
| CD8A | KLRD1 | LC | PP | miR9-5p | RAB27B | LC | M2G |
| KLRD1 | CCR7 | LC | PP | miR9-5p | MICAL3 | LC | M2G |
| CD40LG | GPR29 | LC | PP | miR372-3p | EFNA5 | LC | M2G |
| DEPDC5 | EFNA5 | LC | PP | miR9-5p | ICA1 | LC | M2G |
| CD40LG | CXCL9 | LC | PP | miR9-5p | HIST1H2AE | LC | M2G |
| PPFIBP1 | CCND2 | LC | PP | miR372-3p | KIRREL | LC | M2G |
| CD1B | GPR29 | LC | PP | miR372-5p | FZD2 | LC | M2G |
| ETS1 | CD3E | LC | PP | miR196b-5p | ZBTB10 | LC | M2G |
| GPR29 | TLR2 | LC | PP | miR582-5p | ZFP36L2 | LC | M2G |
| CD8A | CCR7 | LC | PP | miR582-5p | FRMD4A | LC | M2G |
| CD3E | TCF7 | LC | PP | miR582-5p | FZD2 | LC | M2G |
| CD1B | CD28 | LC | PP | miR582-5p | MAP3K13 | LC | M2G |
| P2RY14 | CD38 | LC | PP | miR582-5p | MXD1 | LC | M2G |
| CXCL11 | TLR2 | LC | PP | miR582-5p | NCF2 | LC | M2G |
| CD1B | TLR2 | LC | PP | miR582-5p | NPL | LC | M2G |
| CXCL9 | CD28 | LC | PP | miR582-5p | PDK1 | LC | M2G |
| SPIB | BACH2 | LC | PP | miR582-5p | 8-Sep | LC | M2G |
| CRISP3 | CPA3 | LC | PP | miR582-5p | TLR2 | LC | M2G |
| CSF1 | CXCL11 | LC | PP | miR9-5p | CSF1 | LC | M2G |
| CLEC2D | CCR7 | LC | PP | miR9-3p | PKD2L2 | LC | M2G |
| CD3E | BCL11B | LC | PP | miR582-5p | CRISP3 | LC | M2G |
| CD40LG | CD3E | LC | PP | miR9-3p | APOL6 | LC | M2G |
| RAB27B | SMPD3 | LC | PP | miR9-3p | BCL11B | LC | M2G |
| ABCB9 | CDH12 | LC | PP | miR9-3p | CCR6 | LC | M2G |
| CEACAM8 | CD38 | LC | PP | miR9-3p | CCR7 | LC | M2G |
| CD40LG | LAMP3 | LC | PP | miR9-3p | CDK6 | LC | M2G |
| MAN1A1 | MANEA | LC | PP | miR9-3p | PCDHA5 | LC | M2G |
| CSF1 | CCR7 | LC | PP | miR9-3p | NR4A3 | LC | M2G |
| CSF1 | CLEC7A | LC | PP | miR9-3p | KIRREL | LC | M2G |
| CCL23 | GPR29 | LC | PP | miR9-3p | HHEX | LC | M2G |
| CCL23 | CCR7 | LC | PP | miR582-5p | TREML2 | LC | M2G |
| DENND5B | RAB27B | LC | PP | miR582-3p | STXBP6 | LC | M2G |
| ETS1 | BCL11B | LC | PP | miR582-5p | CD3E | LC | M2G |
| CD40LG | CXCL11 | LC | PP | miR372-5p | LILRA2 | LC | M2G |
| CD40LG | CSF1 | LC | PP | miR372-5p | PDK1 | LC | M2G |
| CD40LG | CLEC7A | LC | PP | miR372-5p | PRR5L | LC | M2G |
| ICA1 | SIK1 | LC | PP | miR372-5p | RALGPS2 | LC | M2G |
| ICA1 | STXBP6 | LC | PP | miR372-5p | REPS2 | LC | M2G |
| TCF7 | CCR7 | LC | PP | miR582-3p | FZD3 | LC | M2G |
| CD1B | CSF1 | LC | PP | miR582-3p | GPC4 | LC | M2G |
| GPR29 | CLEC2D | LC | PP | miR582-3p | KIAA0754 | LC | M2G |
| CD8A | CD40LG | LC | PP | miR582-5p | EFNA5 | LC | M2G |
| ETS1 | TCF7 | LC | PP | miR582-3p | SPIB | LC | M2G |
| REPS2 | BMP2K | LC | PP | miR582-5p | DEPDC5 | LC | M2G |
| CSF1 | CD28 | LC | PP | miR582-5p | BACH2 | LC | M2G |
| miR372-5p | EPN2 | HC | M2G | miR196b-3p | MAP3K13 | LC | M2G |
| miR9-3p | FZD3 | HC | M2G | miR9-5p | BMP2K | LC | M2G |
| miR9-3p | EPN2 | HC | M2G | miR9-5p | ZNF324 | LC | M2G |
| miR9-3p | CREB5 | HC | M2G | miR9-3p | ZBTB10 | LC | M2G |
| miR9-3p | CPA3 | HC | M2G | miR582-5p | CDK6 | LC | M2G |
| miR9-3p | CCND2 | HC | M2G | miR582-5p | CLEC2D | LC | M2G |
| miR582-5p | ZBTB10 | HC | M2G | miR582-5p | CLEC7A | LC | M2G |
| miR582-5p | FZD3 | HC | M2G | miR9-3p | TSHR | LC | M2G |
| miR582-5p | COL8A2 | HC | M2G | miR372-5p | KLRD1 | LC | M2G |
|  |  |  |  | miR582-3p | RAB27B | LC | M2G |

HC^1^: High confidence

LC^2^: Low confidence

PP^3^: protein-protein interaction

M2G^4^: miRNA-target gene pair
