# Supplementary figures and images for: The miR-582/CD1B Axis Is Involved in Regulation of Dendritic Cells and Is Associated with Clinical Outcomes in Advanced Lung Adenocarcinoma
Source: Biomed Res Int. 2020 Mar 17;2020:4360930. doi: 10.1155/2020/4360930 (PMC7103041; doi:10.1155/2020/4360930)

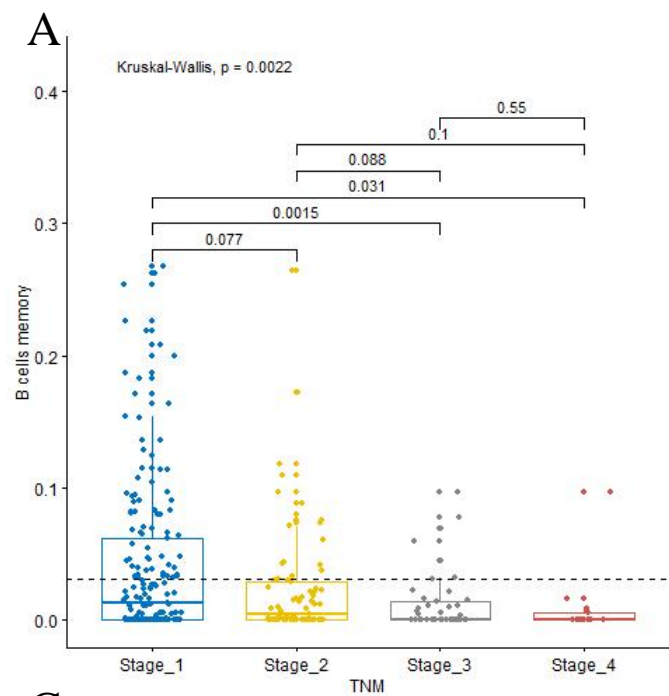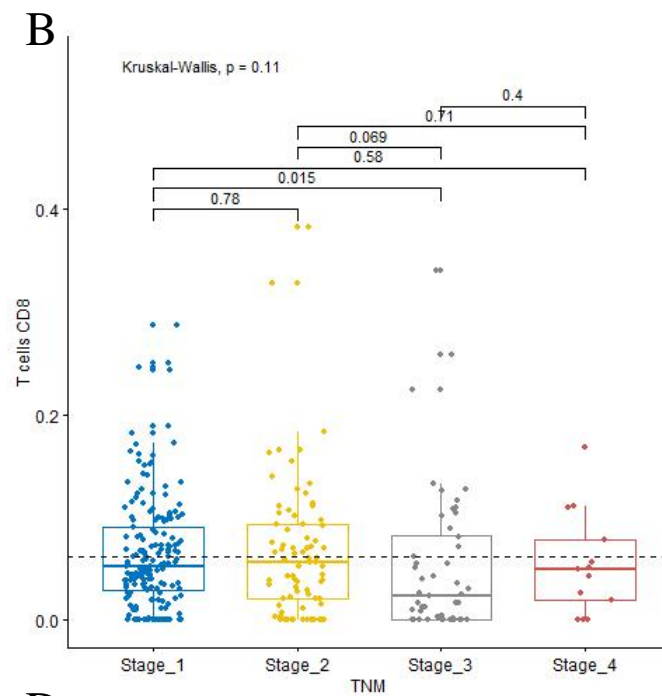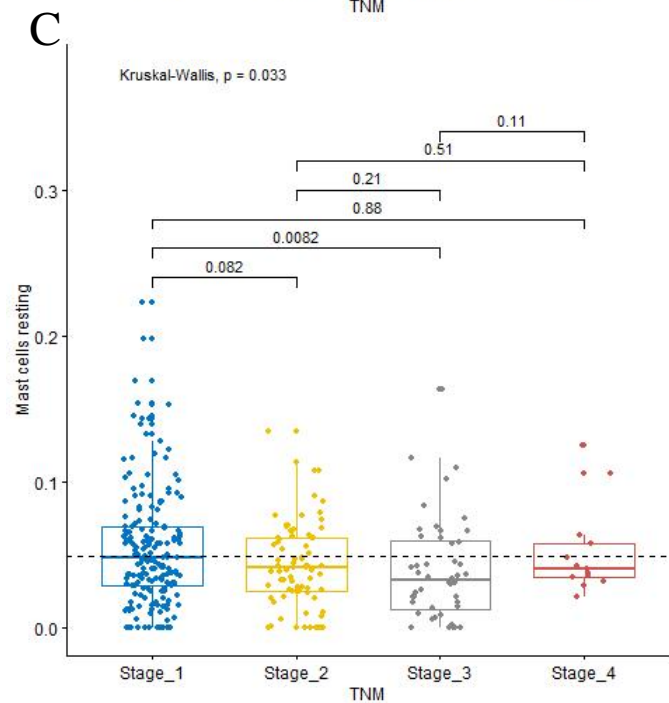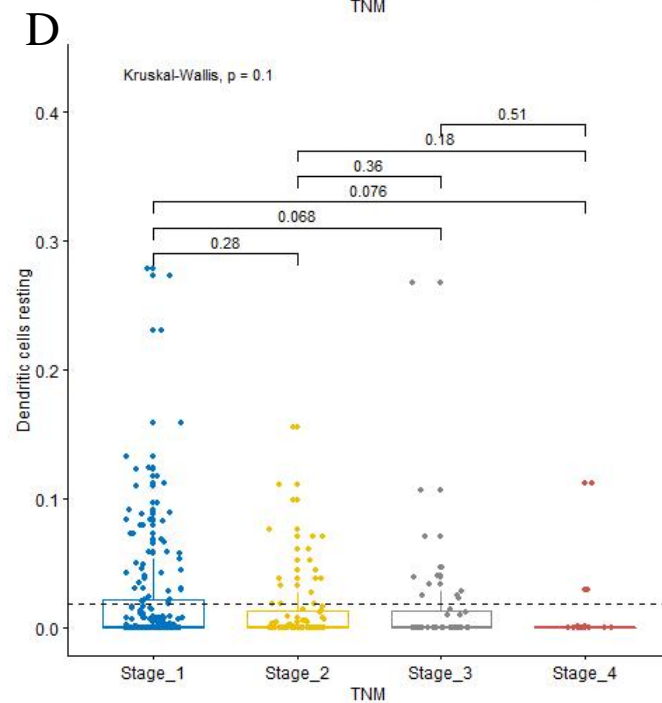

Supplement: Supplementary Materials — 1: the clinical information of 315 samples. Supplementary Materials 2: the fractions of different immune cells of 315 samples. Supplementary Materials 3: the details of miRNA/gene interaction network. Supplementary Figure S1: differences in cell fractions between the four stages. [file 4360930.f1.zip › Figure S1.pdf]
